# Patient perceptions of innovative longitudinal integrated clerkships based in regional, rural and remote primary care: a qualitative study

**DOI:** 10.1186/1471-2296-13-72

**Published:** 2012-07-27

**Authors:** Judith N Hudson, Patricia J Knight, Kathryn M Weston

**Affiliations:** 1 School of Rural Medicine, University of New England, Armidale, NSW 2351, Australia; 2 Graduate School of Medicine, University of Wollongong, Wollongong, NSW, Australia

**Keywords:** Rural medical education, Longitudinal integrated clerkships, Patient-centredness, Patients as stakeholders

## Abstract

**Background:**

Medical students at the University of Wollongong experience continuity of patient care and clinical supervision during an innovative year-long integrated (community and hospital) clinical clerkship. In this model of clinical education, students are based in a general practice ‘teaching microsystem’ and participate in patient care as part of this community of practice (CoP). This study evaluates patients’ perceptions of the clerkship initiative, and their perspectives on this approach to training ‘much-needed’ doctors in their community.

**Methods:**

Semi-structured, face-to-face, interviews with patients provided data on the clerkship model in three contexts: regional, rural and remote health care settings in Australia. Two researchers independently thematically analysed transcribed data and organised emergent categories into themes.

**Results:**

The twelve categories that emerged from the analysis of transcribed data were clustered into four themes: learning as doing; learning as shared experience; learning as belonging to a community; and learning as ‘becoming’. Patients viewed the clerkship learning environment as patient- and student-centred, emphasising that the patient-student-doctor relationship triad was important in facilitating active participation by patients as well as students. Patients believed that students became central, rather than peripheral, members of the CoP during an extended placement, value-adding and improving access to patient care.

**Conclusions:**

Regional, rural and remote patients valued the long-term engagement of senior medical students in their health care team(s). A supportive CoP such as the general practice ‘teaching microsystem’ allowed student and patient to experience increasing participation and identity transformation over time. The extended student-patient-doctor relationship was seen as influential in this progression. Patients revealed unique insights into the longitudinal clerkship model, and believed they have an important contribution to make to medical education and new strategies addressing mal-distribution in the medical workforce.

## Background

A graduate-entry medical school with a mission to develop patient-centred competent graduates with a commitment to regional, rural & remote communities in Australia admitted its first cohort of students in 2007. Strategies to achieve the mission included a positive bias to the admission of students of rural origin, a clinical skills programme including strategies to develop patient-centred care, and early and extended clinical placements in regional, rural and remote settings. As health-care users from these settings are key stakeholders in the outcomes of the medical programme, we aimed to explore ‘patient’ perspectives of the extended involvement of senior medical students in their health care. This followed an earlier study showing regional and rural patients were willing participants in community-based medical education for short-term junior students, and would have accepted more student involvement than actually occurred [1]. The current study aimed to build on this work and address the relative paucity of studies in the medical education literature on the patients’ perspective [2].

Clinical experience in the MBBS programme included a longitudinal integrated clerkship based in general practice, where each student lived, learned and ‘worked’ in a regional, rural or remote community in New South Wales (NSW). During this ‘year-long attachment’, primary health care clinical experience in a range of community settings is integrated with hospital emergency and ward-based patient care. The longitudinal integrated clerkships have been modelled on the Cambridge community-based clinical course in the UK [3] and the Parallel Rural Community Curriculum implemented in several sites in rural South Australia [4,5]. Walters and colleagues have described a typical student week [5]. While some modifications to the model have been necessary according to the context in each of the ten learning hubs in NSW, the core concept is the longitudinal community base in general practice. This is a setting where many of the core values and general skills of other medical disciplines can be developed [6], and first access to acute and chronic care patients (before supervisor review) offers students a range of authentic presentations to develop clinical reasoning skills and patient-centred care.

While longitudinal clerkship students work with several interprofessional hospital and community teams, it is the family practice team in which the student is based that is significant for learner development. Regan-Smith et al. [7] described how to explicitly integrate the learner into an ambulatory care clinic by conceptualising the practice as a ‘microsystem’, and others have shown that extended placement in this teaching microsystem can help students appreciate its importance in the health-care environment [8]. A microsystem has been defined as…*a small group of interdependent people in health care delivery who work together on a regular basis, to provide care to a discrete population of patients*[9], and importantly includes the patient. It comprises two smaller systems: the individual care provider/patient system and, within it, the system of self-care that includes the patient and the sources of information that guide the patient’s choices for his or her care [7]. By integrating the student into the microsystem, the student and preceptor can begin working as a team in the provider/patient system and the student can contribute to the sources of information that guide the patient. Health care is integrated with education [7] and learners can become an asset to the practice, value-adding rather than hindering patient care [10,11].

Lave and Wenger’s situated learning theory, community(s) of practice (CoP) [12,13] provides a theoretical framework that captures the essence of the clerkship pedagogical approach [14]. Participation refers to the process of students being active participants in the practices of social communities and constructing identities in relation to these communities. Wenger describes it as both a kind of action, and a form of belonging [13]. Longitudinal participation in supportive CoPs can shape what the student does, who s/he is, and how s/he interprets this, and can foster increasing student participation and identity transformation over time [13]. Students and supervisors have reported that extended placements can provide continuity of care, curriculum and supervision [15] for students, but how do patients’ perceive this model of clinical education?

The value of involving patients in traditional learning medical student learning environments has long being recognised [16]. Patients in general practice have a positive view towards consulting with junior and senior students, and patient capacity to facilitate student learning is seemingly under-utilised [1,17]. Importantly for this study, patients in general practice held mainly positive views about consulting alone with a student, before seeing their general practitioner (GP) [17]. In the longitudinal integrated clerkships, we had located the student learning in a ‘front-line’ care team that included the patient and wished to gain some understanding of the initiative from the patient perspective: how did patients perceive the learning that arose from this style of clerkship; were they willing to accept a high level of student involvement; and how did the clerkship impact on patients themselves?

## Methods

### Sample

Semi-structured, face-to-face interviews were conducted with patients (N = 13) from three contexts. Patients who had experienced continuity of care from long-term students in their hubs were invited to participate in an interview conducted at their local general practice. Initial attempts to identify patients via the student Clinical Log proved unsuccessful as students were recording individual patient encounters but not generally using the recently introduced facility to record recurrent visits. The numbering system (needed to maintain patient confidentiality) had made this more challenging for students. So students were then invited to identify patients in whose care they had been involved 3 or more times during their longitudinal clerkship, and all those who responded were asked to forward relevant patient names to the practice. To obtain patient perspectives on longitudinal care by an individual student, purposeful sampling was required to recruit patients who had received multiple episodes of care from the student. Practice staff then facilitated contact between the researcher and the patient to issue an invitation to attend for interview. Informed consent was sought by the researcher rather than any health professional, practice staff or student involved in the patient’s health care. While the assistance of current students to identify patients could potentially result in a sample with positive perspectives, it should be noted that the patients’ perspectives were also derived from previous longitudinal students who had no role in sample selection. Ethics approval was obtained from the University of Wollongong Human Research Ethics Committee.

While an attempt was made to sample subjects in a systematic way, the sample tended to involve older patients with chronic conditions. The latter were free to present for interview during practice hours and younger patients tended to present for an acute episode, rather than chronic care. An exception was a young woman presenting for antenatal care, and one couple described their experience with a student when bringing their young grandson for chronic care. As the initiative was only in its second year when the study was undertaken, and there is generally only one student per practice, patients recalled experience with a student from the current or previous year (N = 2 for each region, 5 females and one male in the rural region). Table 1 summarises the patient sample and provides information about the three regions.

**Table 1:** Context of the patient sample

**Locality**	**Patients in sample (Total N = 13)**	**GP services (in addition to ambulatory primary care)**	**Model**
Regional - RA1*	**N = 4**	Nil in-patient services due to large range of hospital specialists	A
Regional major city; non-capital city; 84.5 km south of Sydney; population = 293,782			
Rural - RA2*	**N = 6**	Emergency, obstetric and anaesthetic services	B
Small rural centre; 282 km west of Sydney; population = 8,200			
Remote - RA3*	**N = 3**	Limited in-patient care by GPs due to resident and visiting hospital specialists	C
Large remote centre; 1159 km west of Sydney; population = 21,314			

*RA Classification: http://www.health.gov.au/internet/otd/Publishing.nsf/Content/RA-intro.

### Analysis

The audiotapes of the face-to-face interviews in each location were transcribed for manual theme analysis using inductive qualitative content analysis [18]. Two researchers independently analysed the data and then reached consensus on emergent categories and themes. Data saturation was achieved as the transcripts were analysed. A total of twelve categories emerged and these were clustered into four themes, which outlined the patients’ perspectives of the longitudinal integrated clerkships.

Both researchers were familiar with reported literature on student, preceptor and faculty perspectives of longitudinal integrated clerkships, and anecdotal feedback from the same group of stakeholders. However, they had no prior contact with involved patients, and were not aware of any previous reports of the patients’ perspectives of this model of medical education.

## Results

The twelve categories that emerged from the patient interview transcripts were clustered into four themes. An unexpected outcome was that participating patients described the general practice clinical microsystem as a learning environment not only for students, but also for patients. The patient-student-preceptor relationship triad was highlighted in several themes and categories and was thought to facilitate the participation of both patients and students in delivering the perceived clerkship outcomes.

### Theme 1 – Learning as doing

#### Active participation

Patients highlighted the benefit of learner participation in practice as opposed to book and didactic learning. Students were actively involved with a range of patients.

*You can read anything out of a book…totally different…they’re getting to see people like me but they’re interacting with all age groups* (55-yo-female, remote)

*It’s a good place for the young ones to learn, especially in a busy practice like this because there’s something going on all the time* (65-yo-male, regional)

Active participation by patients was also seen as important. Patients willingly gave consent for student participation. The parallel consulting model [4] which allowed patients to first consult with the student gave opportunity for enhanced student understanding of patients’ perspectives. 

*They’ve got to learn. How are they going to learn if we’re not willing to say yes – at the same time we’re teachers as well* (55-yo-female, remote)

*[Students] said…that they had got a lot from me…they were very fortunate to have met me, it just gave them a better look into what a patient copes with* (55-yo-female, regional)

#### Continuity of care

Patients were positive about continuity afforded by the clerkship model, reporting that the long-term placement allowed student participation in both initial care and follow-up.

*The long-term is a good idea rather than short-term because…you get to know the student and they get to know you…you get the personal touch…because they’ve got the time to get to know exactly what’s going on…as time went on the student got more involved* (55-yo-female, rural)

*They get a totally different experience because…there’s much more follow-through with the hospital* (55-yo-female, rural)

### Theme 2 – Learning as shared experience

Mutual learning resulted from two-way and three-way relationships between doctors, students and patients.

#### The doctor-patient-student relationship

Patients accepted the initial one-on-one consultation with the student, followed by supervision from their doctor. They appreciated being included in open conversations about their care, with a shared understanding arising by the end of the consultation. Patients recognised that their contribution was valued in the 3-way relationship.

*He went right through with her, the questions that she asked, what she had decided should have been the treatment and gave her some suggestions but always while I was present; I appreciated that…yes [felt part of the decision-making process]…nothing was hidden…that’s really important* (45-yo-female, rural)

*They [students] have said things that didn’t quite gel with my experience…you just ask your doctor about it or you query them [student] straight off* (65-yo-male, regional)

#### The doctor-student relationship

The preceptor and student brought their own contribution to a shared understanding of the patient presentation. Patients appreciated the students’ contemporary knowledge.

*They talk a lot together…Dr. will say “what do you think?” and she’ll do the same to the Dr. with different questions* (45-yo-female, rural)

*I love students out of uni…they’re right up with that theoretical knowledge (*45-yo-female, rural)

#### The patient-student relationship

Patients reported that their relationship with students enhanced the quality of patient care. They described students as very personal health-care providers and this facilitated the sharing of perspectives and information.

*I think they are just a lot more aware of the patients* (30-yo-female, rural)

*We had a great talk about everything and I asked him questions and he answered them very professionally…he [student] fulfilled my expectations* (60-yo-female, rural)

### Theme 3 – Learning as belonging

Trust, respect, engagement with community and perceived benefits were seen as features fostering belonging in the community of practice.

#### Trust

Patients advised that trust was a requirement for student participation in their health care. The doctor-patient relationship was the platform for establishing and growing the patients’ trust in the student.

*For me the very important thing is to actually have a doctor I trust working with…it helps in knowing… there’s that interaction that she [student] is going to have with a highly trained doctor [preceptor] (*45-yo-female, rural)

*The patient’s always involved. It’s not like the doctor takes the student out the room…the student actually gets involved…the patient is learning trust…some of these students might be here at the surgery later on in life (*55-yo-female, remote)

#### Respect

Patient respect for their doctor(s) was enhanced by the quality of the ‘preceptorship’ provided to the long-term students.

*What I’ve observed with both doctors, is the way the doctors support the student…probably another spin-off for me is that I’ve got more respect for them…in the way that they’ve actually mentored the two students … it enhances my relationship with the doctor* (45-yo-female, rural)

The mutual respect between student and patient was noted.

*With my grandson—only six but he’s not a dummy…they treated him with respect just like they’d treat an adult patient and to me that’s important* (55-yo-female, remote)

*That’s why I like the students…I may be old and they may be young but they treat me respect and I treat them with respect* (55-yo-female, remote)

#### Community engagement

Patients acknowledged that community involvement beyond healthcare facilitated belonging. This was a function of both the setting and the students’ adaptation to it.

*They seem to get themselves involved in the community which I think is important…they just don’t come here [practice] and go home* (55-yo-female, rural)

*I think country people are maybe more relaxed, more accepting or something* (55-yo-female, rural)

#### Value from accepting students

Inclusion of students in the CoP was seen to enrich the practice and community.

*How important they [students] are to the practice…they’re definitely part of it and I really think the practices need to have them* (55-yo-female, regional)

*We’ve got the centres like the surgery where they’re actually mentoring students… it gives it that high esteem* (45-yo-female, rural)

### Theme 4 – Learning as becoming

#### Student professional identity formation

Patients related how students underwent professional identity formation in the longitudinal learning environment. They believed that students were ‘becoming’ medical practitioners in the context of their rural/regional/ remote community.

*I met her at the beginning of my pregnancy…I went through a few things…you’d come to visit again and she’d be there…it was kind of like she was growing with me* (30-yo-female, rural)

*I think they’re all part of the same team…she is eventually going to be where the doctor is so it’s just basically a line of progression* (30-yo-female, rural)

#### Patient identity transformation

Participants believed that patients also underwent identity transformation in their ‘role as patient’. Consultations involving the student and doctor gave patients greater understanding for shared decision-making and the student was able to contribute to sources of information to guide the patient. Patients felt empowered by being included in the healthcare team, partnering with the doctor to professionally ‘form’ the student. One patient reported feeling valued when she, as well as the doctor, held the knowledge that the student was trying to acquire.

*My own GP said it was something he doesn’t see very often…he wanted to give experience of seeing how she [student] went about diagnosing…he had a quiet word with me…it was very valuable for all of us because it was me talking to the young student doctor…but having the other doctor behind and I knew what the diagnosis was, and he knew* (45-yo-female, rural)

#### Building regional, rural and remote workforce

Patients hoped that the students would become their future medical workforce. Some thought students had already ‘become’ a community doctor. An added bonus was improved access to the patient’s own doctor.

*He’s always good with the students…bringing them up the way that he does it (*60-yo-male, remote)

*I was getting 2 doctors for the price of one…so I can get in and get seen on a regular basis through my treatment with a Doctor who’s been with me from whoa to go* (49-yo-male, regional)

Analysis of patient data from all the three contexts gave rise to common categories and themes. It was interesting to note that some regional and remote patient comments highlighted currentlocal benefits of the longitudinal involvement of the student. One regional (Model A) patient noted how students assisted the doctor.

It’s taken a bit of a load off your GP—they do work pretty hard (65-yo-male, regional)

For remote (Model C) patients who described a ‘revolving door’ of short-term clinicians or students, particularly in the hospital, current relationships with long-term students offered respectful continuity of care.

We have got some very good doctors here but we find that a lot that come through the hospital and they’re in casualty…they might only be here for a month or something but it’s like “you’re country bumpkins” (55-yo-female, remote)

However, several rural patients emphasised the ‘becoming’, i.e. the outcome of the longitudinal integrated clerkships, given the relative shortage of medical workforce in these communities. These rural clerkships were described as a positive strategy with hope for ‘rural return’.

*I think it’s definitely a positive and hopefully when they graduate…it gets them back into the country* (30-yo-female, rural)

## Discussion

All University of Wollongong medical students experience a longitudinal integrated clerkship based in multi-professional general practices linked to GP- or specialist-run community hospitals and community-based healthcare agencies, in regional, rural or remote NSW [14]. While these practices were established to deliver primary and emergency care to catchment populations, they have been shown to be a setting where a system of patient care can be integrated with a system of education [3,4,8].

Research to date on longitudinal placement of students in this ‘teaching microsystem’ has focused on student and preceptor perspectives, rather than those of patients. The current study, attempting to address this deficit, has found that regional, rural and remote patients view the longitudinal integrated clerkships as both patient- and student-centred. This is a desired outcome for an initiative designed to deliver education in the workplace without detracting from the patient experience.

The themes arising from the patient data, namely learning by doing; learning as shared experience; learning as belonging; and learning as becoming, describe the core components of a social-cultural learning theory such as Community of Practice [12,13]. This supports the use of this theoretical framework to inform the clerkship learning environment [14]. However patients felt they, like students, also experienced learning and constructed a new identity in the health care team, by active participation in the CoP.

The longitudinal nature of the community-based clerkships encouraged active participation of patients in their care and student education. Patients gave consent to significant student participation with some gain in autonomy. The student had time to contribute to sources of information that guided the patient’s choices for his or her care. The overt sharing of understanding and decision-making (meaning) encouraged the patients’ sense of belonging in the CoP, assisting them to feel more informed and involved. Just as doctors feel a strong professional identity from being valued as teachers [14], patients felt new status in this role. There was a sense of partnership with the practice in developing and potentially recruiting future medical practitioners for their community. While the trust and respect that patients held for their doctor fostered initial acceptance of the student as a legitimate member of the CoP, it was the ‘two-way’ trust and respect that patients reported that enabled patients and students to progress to central membership.

This is an exciting finding as we endeavour to respond to calls for patient-centred health care reform [19,20]. Sturmberg and colleagues [21] advise that true patient-centred health can only be achieved if all agents in the system adopt ‘the achievement of the patients’ best health experience’ as the shared value for their work. Central to this is learning from patients [21]. Patients reported that this was facilitated in the clerkship model in the time students initially spent consulting independently with the patient, followed by sharing of the patient issue(s) in the student-patient-doctor interaction. 

*Sometime you’re able to talk a bit more at length about different circumstances…I’m not saying that doesn’t happen with your GP too, but having the placements I find absolutely beneficial…you get the feedback from them of what they feel and the questions that they ask you too I find are wonderful…they can relay that to the GP as they come into the room.* (55-yo female, regional)

While the qualitative data were collected by one of the researchers (PJK) who had temporarily worked as a manager of the initiative in all regions (covering maternity leave), she had no further involvement in the program at the time of the study. She had not met any of the participating patients (or students who suggested patients that met the recruitment criteria). She was based at the medical school and had not travelled to the rural or remote region, prior to the study. This researcher took care to collect data free from her own values by allowing participants to answer questions freely. While this generated a considerable amount of less-structured data, it allowed patients to express their opinions about the student clerkship and prior experience with medical students in their region. This researcher did not play a major role in data analysis.

While student assistance was required to recruit suitable patients, consent to participate was sought by the researcher, rather than any providers of patient care. Students provided more patient names than those who gave consent to take part. However, several patients were unable to attend for interview for the limited time the researcher was able to visit each region (this was limited by budgetary constraints). While this limited the sample size and potentially introduced some bias into the sample, a total sample of thirteen patients was thought to achieve data saturation as the same themes arose from all patients. Patients, with mixed backgrounds and levels of education, were all positive about their involvement with long-term students. The sampling method was not thought to recruit only patients who were willing to be involved with students. In these regions, with workforce shortage, it is very rare for patients to refuse student involvement. Without prompting, two patients compared the opportunity students had for one-on-one access to patients in the longitudinal model, comparing it to their prior observation of students’ level of involvement in the hospital, as follows:

*In hospital, the students mostly they come around with the doctor…basically what they had to do was write out a case history—know what I mean..about four [students at a time]* (65-yo-male, regional)

## Conclusions

Patients revealed unique insights into the longitudinal clerkship model, and believed they have an important contribution to make to medical education and new strategies addressing mal-distribution in the medical workforce. The longitudinal patient-student-preceptor triad which facilitated active participation by all was seen as central to clerkship outcomes. Patients reported that students learned, becoming ‘assistant physicians’ over the academic year, making a positive contribution to patient-care. Future research should continue to focus on patients to explore how different models of patient involvement in medical education impact on patient outcomes. In the light of this study it would be interesting to follow-up on the value of patient-professional relationships that include students, for patient personal autonomy. Mountouxe [22] advised that medical education should further understand the process through which we develop our identities. Greater insight into the process of how longitudinal integrated clerkships impact on student professional identity development, may enable us to tailor the learning experience to develop the professional identities that doctors require for future practice.

## Competing interests

The authors declare that they have no competing interests.

## Authors’ contributions

JNH, PJK and KMW designed and managed all aspects of the research. JNH and KMW supervised the collection of the qualitative data, gathered by PJK. JNH and KMW did most of the analysis of the data, with limited input from PJK. JNH wrote the first draft of the paper and contributed to later drafts. PJK and KMW contributed to later drafts of the paper. All authors approved the final version of the paper.

### Funding

The Department of Health and Ageing, Commonwealth Government of Australia provided funding support for the project.

## Pre-publication history

The pre-publication history for this paper can be accessed here:

http://www.biomedcentral.com/1471-2296/13/72/prepub
